# Supplementary material for: Student Perceptions of Substance Use Disorder Stigma as a Factor for Health Disparities: A Mixed-Methods Study
Source: Pharmacy (Basel). 2023 Jul 1;11(4):112. doi: 10.3390/pharmacy11040112 (PMC10366849; doi:10.3390/pharmacy11040112)
Supplement: Supplementary file 1 [file pharmacy-11-00112-s001.zip › pharmacy-2368608-supplementary.pdf]

# Supplementary Material

**Table S1.** Student Respondent Characteristics.

| | n | % |
|---|---|---|
| Age (years) | 25 (SD: 4) | |
| Sex | | |
| Male | 74 | 20 |
| Female | 292 | 79 |
| Prefer not to answer | 3 | 1 |
| Hispanic, Latino, or Spanish Origin | | |
| Yes | 12 | 3 |
| No | 357 | 97 |
| Race | | |
| American Indian or Alaska Native | 3 | 1 |
| Asian Indian | 21 | 6 |
| Black or African American | 55 | 15 |
| Chinese | 10 | 3 |
| Filipino | 5 | 1 |
| Japanese | 2 | 1 |
| Other Asian | 5 | 1 |
| Other Pacific Islander | 1 | 0 |
| Some other race | 12 | 3 |
| Vietnamese | 4 | 1 |
| White | 248 | 68 |
| College | | |
| Dentistry | 3 | 1 |
| Graduate Health Sciences | 21 | 6 |
| Health Professions | 42 | 11 |
| Medicine | 70 | 19 |
| Nursing | 51 | 14 |
| Pharmacy | 182 | 49 |

**Table S2.** Student respondent comparisons among reported college and selected survey questions.

| Dependent Variable | What College are you in? | What College are you in? | Sig. |
|---|---|---|---|
| Stigma currently exists in the healthcare field. | Dentistry | Graduate Health Sciences | .879 |
| | | Health Professions | .809 |
| | | Medicine | .991 |
| | | Nursing | 1.000 |
| | | Pharmacy | .991 |
| | Graduate Health Sciences | Dentistry | .879 |
| | | Health Professions | 1.000 |
| | | Medicine | .773 |
| | | Nursing | .134 |
| | | Pharmacy | .699 |
| | Health Professions | Dentistry | .809 |

| | | | |
|---|---|---|---|
| | | Graduate Health Sciences | 1.000 |
| | | Medicine | .327 |
| | | Nursing | .010 |
| | | Pharmacy | .179 |
| | Medicine | Dentistry | .991 |
| | | Graduate Health Sciences | .773 |
| | | Health Professions | .327 |
| | | Nursing | .536 |
| | | Pharmacy | 1.000 |
| | Nursing | Dentistry | 1.000 |
| | | Graduate Health Sciences | .134 |
| | | Health Professions | .010 |
| | | Medicine | .536 |
| | | Nursing | .375 |
| | Pharmacy | Dentistry | .991 |
| | | Graduate Health Sciences | .699 |
| | | Health Professions | .179 |
| | | Medicine | 1.000 |
| | | Nursing | .375 |
| Stigma can lead to lower quality care provided to patients with substance use disorders. | Dentistry | Graduate Health Sciences | .996 |
| | | Health Professions | .777 |
| | | Medicine | .999 |
| | | Nursing | 1.000 |
| | | Pharmacy | 1.000 |
| | Graduate Health Sciences | Dentistry | .996 |
| | | Health Professions | .490 |
| | | Medicine | 1.000 |
| | | Nursing | .985 |
| | | Pharmacy | .989 |
| | Health Professions | Dentistry | .777 |
| | | Graduate Health Sciences | .490 |
| | | Medicine | .059 |
| | | Nursing | .027 |
| | | Pharmacy | .005 |
| | Medicine | Dentistry | .999 |
| | | Graduate Health Sciences | 1.000 |
| | | Health Professions | .059 |
| | | Nursing | .995 |
| | | Pharmacy | .996 |
| | Nursing | Dentistry | 1.000 |
| | | Graduate Health Sciences | .985 |
| | | Health Professions | .027 |
| | | Medicine | .995 |
| | | Nursing | 1.000 |

| | | | |
|---|---|---|---|
| | Pharmacy | Dentistry | 1.000 |
| | | Graduate Health Sciences | .989 |
| | | Health Professions | .005 |
| | | Medicine | .996 |
| | | Nursing | 1.000 |

**Table S3.** Student respondent comparisons among reported race and selected survey questions.

| | | Sum of Squares | df | Mean Square | F | Sig. |
|---|---|---|---|---|---|---|
| Stigma currently exists in the healthcare field. | Between Groups | 6.362 | 10 | .636 | 1.277 | .242 |
| | Within Groups | 175.825 | 353 | .498 | | |
| | Total | 182.187 | 363 | | | |
| Stigma can lead to patients' not receiving the appropriate care for a substance use disorder. | Between Groups | 11.476 | 10 | 1.148 | 2.362 | .010 |
| | Within Groups | 171.524 | 353 | .486 | | |
| | Total | 183.000 | 363 | | | |
| Stigma can lead to lower quality care provided to patients with substance use disorders. | Between Groups | 12.070 | 10 | 1.207 | 2.106 | .023 |
| | Within Groups | 202.932 | 354 | .573 | | |
| | Total | 215.003 | 364 | | | |
